# Protein phosphatase 2A regulation of GABA_*B*_ receptors normalizes ischemia-induced aberrant receptor trafficking and provides neuroprotection

**DOI:** 10.3389/fnmol.2022.1015906

**Published:** 2022-10-13

**Authors:** Mohammad Hleihil, Karthik Balakrishnan, Dietmar Benke

**Affiliations:** ^1^Institute of Pharmacology and Toxicology, University of Zurich, Zurich, Switzerland; ^2^Neuroscience Center Zurich, University of Zurich and ETH Zürich, Zurich, Switzerland; ^3^Drug Discovery Network Zurich, Zurich, Switzerland

**Keywords:** protein phosphatase 2A, GABA_*B*_ receptor, interfering peptide, cerebral ischemia, excitotoxicity, neuroprotection

## Abstract

One major factor regulating the strength of GABA_*B*_ receptor signaling and thereby neuronal excitability is the dynamic control of their cell surface expression. GABA_*B*_ receptors are constitutively internalized and recycled back to the plasma membrane to maintain a stable number of receptors at cell surface for appropriate signaling. Protein phosphatase 2A (PP2A) dependent dephosphorylation of serine 783 (S783) in the GABA_*B*2_ subunit is a key event for downregulating GABA_*B*_ receptor cell surface expression particularly under conditions associated with excitotoxicity. Here, we investigated the role of PP2A in regulating GABA_*B*_ receptor cell surface expression under physiological and excitotoxic conditions. For this purpose, we developed an interfering peptide (PP2A-Pep) that inhibits the interaction of GABA_*B*_ receptors with PP2A. Using cultured cortical neurons, we found that PP2A downregulates GABA_*B*_ receptor cell surface expression by inhibiting recycling of the receptors and thereby promoting degradation of the receptors. Inhibition of the GABA_*B*_ receptor/PP2A interaction by PP2A-Pep in cultured cortical neurons restored GABA_*B*_ receptor cell surface expression after excitotoxic stress and inhibited progressing neuronal death even when added 48 h after the insult. To explore the therapeutic potential of PP2A-Pep, we further analyzed effect of PP2A-Pep in the middle cerebral artery occlusion (MCAO) mouse model of cerebral ischemia. Incubation of brain slices prepared from MCAO-treated mice with PP2A-Pep restored normal GABA_*B*_ receptor expression and GABA_*B*_ receptor-mediated inhibition, reduced ischemic-induced overexcitability of neurons, and prevented neuronal death in the ischemic penumbra. This data illustrates the crucial role of regulating GABA_*B*_ receptor phosphorylation by PP2A for controlling neuronal inhibition and excitability. The results further suggest that interfering with the GABA_*B*_ receptor/PP2A interaction is a promising strategy for the development of specific therapeutic interventions to treat neurological diseases associated with a disturbed excitation/inhibition balance and downregulation of GABA_*B*_ receptors.

## Introduction

The GABA_*B*_ receptor is one of the key regulators of neuronal excitability by mediating sustained inhibition (Chalifoux and Carter, 2011). They are expressed at pre- and postsynaptic sites in most neurons and are involved in virtually all fundamental brain functions. GABA_*B*_ receptors are G protein-coupled receptors (GPCRs) composed of the two subunits, GABA_*B*1_ and GABA_*B*2_ (Bettler et al., 2004; Gassmann and Bettler, 2012; Terunuma, 2018). Binding of GABA to the receptors activates G_*i/o*_ proteins which in turn regulate several downstream effectors. GABA_*B*_ receptor-induced neuronal inhibition is mainly mediated by hyperpolarization of the neuronal plasma membrane via enhancing potassium influx through G protein-coupled inwardly rectifying potassium (GIRK or K_*ir*_3) channels (Gähwiler and Brown, 1985; Luscher et al., 1997; Chalifoux and Carter, 2011) and by inhibition of neurotransmitter release via suppression of voltage-gated Ca^2+^ channels (Mintz and Bean, 1993; Santos et al., 1995; Chen and van den Pol, 1998; Bussieres and El Manira, 1999; Chalifoux and Carter, 2011).

A crucial determinant for the strength of GABA_*B*_ receptor-mediated inhibition is the number of receptors available at the cell surface for signaling. This is regulated by a precise balance of distinct trafficking mechanisms including receptor exocytosis, endocytosis, recycling, and degradation (Benke, 2010). GABA_*B*_ receptors are constitutively internalized via clathrin- and dynamin-dependent endocytosis. Internalized receptors are rapidly recycled back to the cell surface or targeted to lysosomes for degradation. Under pathological conditions associated with neuronal overexcitation, the balance of recycling and degradation of the receptors is shifted toward degradation, thereby downregulating cell surface expression of GABA_*B*_ receptors (Guetg et al., 2010; Maier et al., 2010; Terunuma et al., 2010; Kantamneni et al., 2014). This is a very detrimental mechanism since one fundamental function of GABA_*B*_ receptors is to counter-balance neuronal hyperexcitability to prevent neurons to enter a state of overexcitation that may lead to excitotoxic death.

Two key events have been identified causing the aberrant sorting of the receptors to lysosomes for degradation. (1) Sustained activation of glutamate receptors induces Ca^2+^/calmodulin-dependent protein kinase II (CaMKII)-mediated phosphorylation of serine 867 of GABA_*B*1_ (Guetg et al., 2010). This phosphorylation event serves as a signal for K63-linked ubiquitination of the receptor by the E3-ligase Mind-bomb 2 (MIB2) and sorts the receptors to lysosomes for degradation (Zemoura et al., 2019). (2) Activation of protein phosphatase 2A (PP2A) dephosphorylates serine 783 of GABA_*B*2_. This most likely affects constitutive internalization and recycling of the receptors (Terunuma et al., 2010).

There is mounting evidence that PP2A-mediated dephosphorylation of GABA_*B*2_(S783) and the associated reduction of cell surface GABA_*B*_ receptors plays, beside in cerebral ischemia, an important role in neurological diseases such as addiction (Maeda et al., 2006; Padgett et al., 2012; Hearing et al., 2013) and depression (Lecca et al., 2016). In either case, blocking PP2A activity with specific inhibitors reduced disease symptoms in mouse models of the diseases, indicating PP2A as promising drug target. However, systemic inhibition of PP2A activity is not a favored option for a therapeutic intervention because PP2A is involved in a variety of physiological functions and global inhibition of PP2A is expected to be associated with severe side effects. Therefore, a more targeted strategy addressing specifically the GABA_*B*_ receptor/PP2A interaction might be a promising solution to this problem.

In this study, we aimed at clarifying in greater detail the trafficking pathway(s) affected by enhanced PP2A-dependent dephosphorylation of GABA_*B*2_(S783) and exploring the potential therapeutic benefit of targeting specifically the GABA_*B*_ receptor/PP2A interaction. For this, we developed a short interfering peptide (PP2A-Pep) able to block the interaction of PP2A with GABA_*B*_ receptors. We used PP2A-Pep for analyzing the pathway affected by PP2A in untreated and glutamate-stressed cultured neurons and tested it for its effects on GABA_*B*_ receptor expression, function, and neuroprotective activity in the MCAO mouse model of cerebral ischemia.

## Materials and methods

### Animals

Animal experiments were performed according to the national guidelines of the Swiss Federal act on animal protection. All animal experiments were approved by the Zurich cantonal veterinary office, Zurich, Switzerland (license ZH152/16, ZH011/19, and ZH031/16).

Middle cerebral artery occlusion experiments were performed unblinded using 9–12 weeks old C57BL/6J male mice. Animals were housed up to five per cage with a standard 12 h light/12 h dark cycle. Food and water were available *ad libitum*. The mice were randomly assigned to MCAO or sham operation. Primary neuron/glia cultures were prepared from E18 embryos of Wistar rats. All animals used in this study were purchased from ENVIGO (Netherlands).

### Antibodies

The following primary antibodies were used in this study: mouse anti-EEA1 (1:25, BD Biosciences Cat# 610456), mouse anti-GABA*_*B*2_* (1:400, Abcam ab181736), rabbit anti-GABA*_*B*2_* (1:400, Abcam ab75838), rabbit anti-GABA_*B*2_N (1:250, custom-made by GeneScript, Benke et al., 2002), mouse anti-HA-tag (1:400, Sigma Aldrich, H9658), rabbit anti-NeuN (1:400, Millipore ABN78), rabbit anti-PP2A (1:400, MyBioSource MBS858915), mouse anti-Rab4 (1:25, BD Biosciences Cat# 610888) and rabbit anti-Rab7 (1:50, Abcam ab137029). Secondary antibodies: donkey anti-rabbit AlexaFluor Plus 488 (1:2000, Thermo Fisher Scientific A32790), donkey anti-mouse AlexaFluor Plus 555 (1:2000, Thermo Fisher Scientific A32773), goat anti-rabbit AlexaFluor Plus 800 (1:2000, Thermo Fisher Scientific A32735), and AlexaFluor 488-conjugated streptavidin (1:500, Jackson ImmunoResearch 016-540-084). All normal laboratory chemicals used were purchased from Sigma-Aldrich.

### Interfering peptide

The interfering peptide (PP2A-Pep) contains a sequence derived from the intracellularly located C-terminal domain of GABA_*B*2_ (FQFTQNQKKEDSKTSTSV). For controls, a peptide (Ctrl-Pep) containing the same amino acids but in a random sequence (QKFSVNTFQEKDTKSQTS) was used. The peptides were rendered cell-permeable by tagging them at their N-terminus with a peptide sequence derived from the Rabies virus glycoprotein followed by 9 arginine residues (YTIWMPENPRPGTPCDIFTNSRGKRASNGGGG-RRRRRRRRR) (Kumar et al., 2007). Peptides were custom-synthesized by Pepmic Co., Ltd, Suzhou, China with an N-terminal biotin for detection and used at a concentration of 10 μg/ml throughout the experiments.

### Primary cortical neuron/glia co-cultures

Cortical neurons were prepared from E18 Wistar rat embryos as described previously (Buerli et al., 2007). Briefly, cortices from embryos were dissected and washed with 5 ml PBGA buffer [PBS, 10 mM glucose, antibiotic-antimycotic solution (1:100, Invitrogen)]. The tissue was digested in papain solution (0.5 mg/ml papain, 1 mg/ml BSA, 10 μg/ml DNAse I and 10 mM glucose in PBS) for 15 min at 37°C and thereafter washed twice with 2 ml Dulbecco’s Modified Eagle’s Medium (DMEM) containing 10% fetal calf serum (FCS) and 1:100 antibiotic-antimycotic solution (complete DMEM). Tissue was triturated with a fire-polished Pasteur pipette and cells were plated at a density of 60,000 cells/ml onto poly-L-lysine coated 18 mm coverslips placed in a 12 well culture plate. After incubation overnight in complete DMEM at 37°C and 5% CO_2_, the complete DMEM medium was exchanged for NU-medium (MEM, supplemented with 15% Nu-serum, 2% B27 supplement, 15 mM HEPES pH 7.1, 0.45% glucose, 1 mM sodium pyruvate, 2 mM GlutaMAX) and cells were incubated at 37°C and 5% CO_2_ for further 11–15 days.

### Transfection of cultured neurons

For expression of recombinant proteins, cultured neurons were transfected with 1 μg of the respective plasmid DNA by magnetofection using Lipofectamine 2000 (Invitrogen) and CombiMag (OZ Biosciences) at 7–12 DIV following a previously described protocol (Buerli et al., 2007). Neurons were analyzed 48–96 h after transfection. The following cDNAs in expression vectors were used: EGFP (gift from Dr. Shiva Tyagarajan), GABA_*B*1_ (Kaupmann et al., 1997), HA-tagged GABA_*B*2_ (Kaupmann et al., 1998), HA-tagged GABA_*B*2_(S783A) (mutation inserted by Genescript), HA-tagged GABA_*B*2_(S783D) (mutation inserted by Genescript) and GABA_*B*2_(BBS) tagged with the HA-tag and the minimum α-bungarotoxin binding site (BBS) (attached to the extracellularly located N-terminus using standard cloning techniques), HA-tagged PP2A-C (catalytic subunit of PP2A, Evans et al., 1999), and HA-tagged PP2A-C(L199P) (inactive mutant of PP2A-C, Evans et al., 1999).

### Immunofluorescence staining of cell cultures

#### Total staining

Coverslips were rinsed in PBS and transferred to 4% PFA/4% sucrose solution for 15 min at room temperature. After fixation, cells were washed with PBS and permeabilized by incubation for 12 min in PBS containing 0.2% Triton X-100. Then, cultures were incubated with primary antibodies diluted in PBS supplemented with 10% normal donkey serum (NDS) in a humidity chamber overnight at 4°C. After incubation, cells were washed three times with PBS and incubated with secondary antibody diluted in PBS, 10% NDS for 1h at room temperature. Finally, coverslips were washed with PBS, dried, and mounted upside down on glass slides (SuperFrost Plus, Thermo Scientific) with DAKO fluorescent mounting medium (Agilent Technologies).

#### Cell surface staining of GABA_*B*_ receptors

Coverslips were washed twice with pre-cooled Krebs solution (2 mM CaCl_2_, 2 mM MgCl_2_, 5 mM KCl, 30 mM glucose, 25 mM, 119 mM NaCl, HEPES pH 7.4) and then incubated on ice for 2 h with GABA_*B*2_N antibody directed against the extracellularly located N-terminus of GABA_*B*2_ (1:250 dilution in Krebs solution containing 10% NDS). The coverslips were washed three times for 5 min with ice cold Krebs solution and incubated for 1 h with donkey anti-rabbit secondary antibody in PBS/10% NDS. Cells were fixated with 4% PFA/4% sucrose for 15 min at room temperature. After fixation, the cells were washed twice with PBS, dried and mounted on glass slides with DAKO fluorescent mounting medium.

### *In situ* proximity ligation assay (*in situ* proximity ligation assay)

We used the highly sensitive *in situ* proximity ligation assay (PLA) (Soderberg et al., 2006; Leuchowius et al., 2010) to test for the interaction of GABA_*B*_ receptors with PP2A and for their colocalization with the endosomal marker proteins EEA1, Rab4, and Rab7. Cultures were washed with PBS, fixated with 4% PFA/4% sucrose for 20 min at room temperature and permeabilized with 0.2% Triton X-100 in PBS for 10 min. Neurons were then incubated overnight at 4°C with the appropriate pair of antibodies for GABA_*B*2_ and either PP2A, EEA1, Rab4 or Rab7. Subsequently, *in situ* PLA was performed using the Duolink kit (Sigma Aldrich) exactly according to the manufacturer’s instructions. AlexaFluor Plus 488-conjugated secondary antibodies were added during the final polymerization step to stain for total GABA_*B*_ receptor expression.

### Induction of excitotoxicity, peptide treatment, and quantification of neuronal loss *in vitro*

Excitotoxic conditions were induced by incubating cultures with 50 μM glutamate (Sigma Aldrich) for 1 h. For this, half of the medium (1 ml) was removed from the cultures and stored at 37°C and 5% CO_2_. Then, cultures were treated with 50 μM glutamate (Sigma Aldrich) for 1 h at 37°C and 5% CO_2_, washed thereafter with PBS and further incubated for 12–16 h in the saved original conditioned culture medium supplemented with PP2A-Pep (10 μg/ml) or with Ctrl-Pep (10 μg/ml) before testing.

To determine the neuroprotective activity of PP2A-Pep, PP2A-Pep, or Ctrl-Pep were added at different timepoints after the glutamate stress (3, 6, 9, 12, 24, and 48 h) and cells were analyzed 24 h following each timepoint. Hence, neurons were exposed to the peptides for equal duration.

To test for neuron survival, cultures were stained with an antibody directed against the neuron-specific marker protein NeuN, followed by staining with AlexaFluor Plus 488-conjugated secondary antibodies. The total number of cells was determined by counting the cell nuclei stained with DAPI (1:2000) added with the secondary antibody. Surviving neurons were quantified by counting the number of neurons in relation to the number of DAPI positive nuclei (mainly glia cells) using ImageJ software.

### Live cell imaging for tracking GABA_*B*_ receptor internalization

Monitoring GABA_*B*_ receptor internalization by life cell imaging of GABA_*B*_ receptors tagged with the α-bungarotoxin binding site (BBS) was performed as described previously by Hannan et al. (2013) with minor modifications. The minimum sequence for the α-bungarotoxin binding site (BBS, WRYYESSLEPYPD) along with two HA-tag sequences (YPYDVPDYA-YPYDVPDYA) were attached to the extracellularly located N-terminus of GABA_*B*2_ [GABA_*B*2_(BBS)] using standard cloning techniques. Cultures were transfected at DIV 7–9 with GABA_*B*2_(BBS) together with EGFP (for fast identification of transfected neurons) and cultured for further 72–96 h. For live cell imaging, cultures were washed with ice-cold Krebs solution (140 mM NaCl, 4.7 mM KCl, 1.2 mM MgCl_2_, 2.5 mM CaCl_2_, 11 mM glucose, and 5 mM HEPES pH 7.4), preincubated with 1 mM D-tubocurarin (Sigma-Aldrich) for 5 min to block endogenous nicotinic acetylcholine receptors containing the α7 subunit and then incubated with 3 μg/ml AlexaFlour 555-conjugated α-bungarotoxin (BTX, Invitrogen, B35451) for 15 min on ice to label all GABA_*B*_ receptors containing GABA_*B*2_(BBS) expressed at the cell surface (incubation at low temperature inhibited internalization of the receptors). Following three washes with ice-cold Krebs solution to remove excess BTX, neurons were transferred to the imaging chamber with warm Krebs solution at room temperature to permit internalization of the receptors. Cells expressing EGFP were located as quickly as possible, and settings were optimized for acquiring BTX florescence at time zero (t_0_). Imaging of a single plane was performed at Nyquist sampling and eight times averaging to increase the signal/noise ratio. Glutamate (50 μM) and PP2A-Pep (10 μg/ml) were added after adjusting the imaging settings directly to the imaging chamber. Cells were captured at the indicated timepoints without modifying the acquisition setting.

The rate of GABA_*B*_ receptor internalization was assessed by measuring the mean fluorescence intensity at the cell surface at every timepoint. Measurements were normalized to the mean cell surface membrane fluorescence obtained at t_0_ timepoint.

### Middle cerebral artery occlusion

Middle cerebral artery occlusion (MCAO) was performed on naïve adult male C57Bl/6J mice as described previously (Hleihil et al., 2021). Briefly, mice were anesthetized with isoflurane and analgesia was provided by injection of buprenorphine and lidocaine. The left common carotid artery was exposed through a midline incision in the neck and unilateral MCAO was conducted by inserting a 7-0 silicone rubber-coated monofilament (Doccol Corp., Sharon, MA, United States, 701956PK5Re) to occlude the middle cerebral artery. During the occlusion, the mice were kept at 37°C in a ventilated recovery chamber. After exactly 60 min, the filament was withdrawn to allow reperfusion. After additional 60 min in the recovery chamber the mice were euthanized for the *ex vivo* analysis. In sham operated mice, the filament was inserted into left middle cerebral artery and immediately withdrawn to allow instant reperfusion. The Bederson score for testing impaired neurological/motor function (Schaar et al., 2010) was used to verify successful MCAO surgery.

### Electrophysiology on acute brain slices

Patch clamp electrophysiology was performed on acute brain slices obtained from MCAO or sham operated mice. After 60 min reperfusion, brains were extracted and cut into 300 μm coronal cortical slices using a vibratome with a horizontally oscillating blade (HM 650, Microm). Slices were then incubated in oxygenated artificial cerebrospinal fluid (ACSF, 125 mM NaCl, 2.5 mM KCl, 1.25 mM NaH_2_PO_4_, 25 mM NaHCO_3_, 1 mM MgCl_2_, 2 mM CaCl_2_, and 25 mM glucose, pH 7.4, osmolarity 315 mOsm) supplemented with 10 μg/ml PP2A-Pep or Ctrl-Pep at 34°C for 30 min and then at room temperature until analysis. Recordings were performed 2–6 h after peptide treatment at 32–34°C from cortical pyramidal neurons, which were identified by their shape, large soma and firing patterns.

To measure baclofen-induced currents, borosilicate glass patch pipettes (3.5–4.5 mΩ) were filled with potassium gluconate based intracellular solution consisting of 135 mM potassium gluconate, 2 mM NaCl, 4 mM KCl, 4 mM EGTA, 10 mM HEPES, 4 mM Mg-ATP, and 0.3 mM Na_3_GTP, pH 7.3, 295 mOsm. Neurons were held at –50 mV and GABA_*B*_ receptor-mediated currents were measured as a response to bath application of 100 μM baclofen (Tocris Bioscience). For some neurons, GABA_*B*_ receptor-mediated GIRK currents were confirmed by application of the GABA_*B*_ receptor antagonist CGP36742 (10 μM, Sigma Aldrich).

Neuronal excitability was determined in current-clamp experiments using 135 mM potassium gluconate intracellular solution. Throughout all experiments, cells were maintained at their original membrane potential. To measure the current-voltage (I–V) curve, a series of 50 pA current steps were performed (from –350 to 450 pA, 250 ms duration). The input resistance was calculated from the voltage responses after applying a 150 pA hyperpolarizing current pulse in the current clamp mode. The resting membrane potential (*V*_*m*_) was determined immediately after acquiring the whole cell mode and the firing threshold (*V*_*th*_) was determined by injection of depolarization current steps (5 pA, 250 ms) until the first action potential was generated.

Recordings were excluded from the analysis if either the series resistance (Rs) varied by more than 20% throughout the experiment or the resting membrane potential was depolarized more than -55 mV. Currents were filtered at 5 kHz and digitized at 20 kHz. All recordings were performed using a Multiclamp 700B amplifier, acquired with Digidata 1550A, and analyzed offline using Clampfit 10.5 (Molecular Devices).

### *Ex vivo* peptide administration and immunofluorescence staining of brain sections

Brain sections obtained from MCAO or sham operated mice were incubated with PP2A-Pep to test its neuroprotective activity and its ability to restore GABA_*B*_ receptor expression. Brains were handled and cut similar to acute brain slice preparation procedure for electrophysiology (see above) with small modifications. Brains were sliced into 90 μm thick coronal using a vibrotome, followed by incubation in a modified 12-well culture plate containing ACSF with constant oxygenation (95% O_2_ and 5% CO_2_). Peptides (10 μg/ml) were added to each well simultaneously and slices were incubated at room temperature for 6 h with constant oxygenation. Subsequently, the slices were fixated with 4% PFA for 60 min at room temperature. Staining was performed with free-floating sections. Sections were incubated with rabbit anti-NeuN and mouse anti-GABA_*B*2_, diluted in Tris-Triton solution (50 mM Tris pH 7.4, 150 mM NaCl, 0.2% Triton X-100) containing 2% NDS, overnight at 4°C. After three washes for 10 min with Tris-Triton, sections were incubated with secondary antibodies diluted in Tris-Triton/2% NDS for 1 h at room temperature [donkey anti-mouse AlexaFluor Plus 555, donkey anti-rabbit AlexaFluor Plus 647, and AlexaFluor 488-conjugated streptavidin (to stain for biotin-tagged peptides)]. After five washes with Tris-Triton, sections were placed onto glass slides (SuperFrost Plus, Thermo Scientific), dried at room temperature and coversliped with DAKO fluorescence mounting medium.

### Confocal laser scanning microscopy and image analysis

Stained brain sections and neuron/glia cultures were analyzed by confocal laser scanning microscopy (LSM 700 or LSM 800, Zeiss) using 40x (1.4 NA) or 63x (1.4 NA) plan-apochromat oil differential interference contrast objectives and sampled sequentially in 4–5 z-planes according to the Nyquist criterions. For some experiments an optical zoom was applied, and sampling parameters were optimized accordingly. For quantitative analysis, all images belonging to an experiment were recorded with identical settings. Analysis was performed after merging the optical planes into a single image.

Images were recorded using the Zen 2.6 software (Zeiss) and further data analysis was conducted with ImageJ Fiji (Schindelin et al., 2012). For analysis of cell surface staining, the outer and the inner border of the plasma membrane were outlined, and the mean fluorescence intensity was measured. Then the value of the inner surface was subtracted from the value of the outer surface. For analysis of total staining, the outer border of each cell was outlined, and the mean fluorescence intensity was measured. The quantification of *in situ* PLA signals was performed using the ImageJ plug-in *Analyze Particles*. A Gaussian blur filter was applied (sigma = 1), followed by background subtraction (Rolling Ball radius = 30). The build-in Moments algorithm was applied to determine the signal threshold for all images. For each experiment, images were reviewed separately, and the noise tolerance was set to ensure that all PLA signals were counted. The PLA signals of neurons were normalized to the total expression level of GABA_*B*2_ and to the area analyzed.

### Statistics

The data are depicted as means ± SD. Statistical evaluation of data sets were performed with either one-way or two-way analysis of variance (ANOVA) followed by Tukey’s *post hoc* tests using GraphPad Prism 8. D’Agostino-Pearson test and QQ plots were employed to test for normal or lognormal distributions. In case of significant deviation from homoscedasticity Welch and Brown Forsythe variations of ANOVA was used. Differences were considered statistically significant when *p* < 0.05. The statistical test used is indicated in the respective figure legends.

## Results

### PP2A regulates GABA_*B*_ receptor expression

**FIGURE 1:**
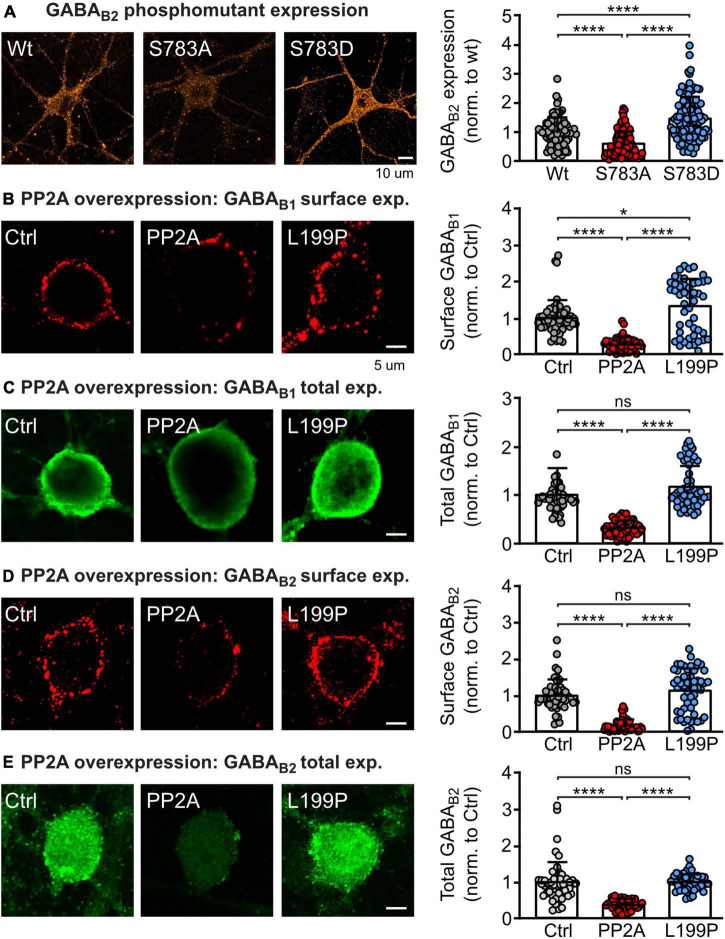
PP2A regulates GABA_B_ receptor expression. **(A)** Phosphorylation/dephosphorylation of GABA_B2_(S783) affects expression of GABA_B_ receptors. Neurons were transfected either with wild type HA-tagged GABA_B2_, a HA-tagged GABA_B2_ mutant with inactivated S783 phosphorylation site 783 (S783A) or with a mutation mimicking phosphorylation of this site (S783D) along with wild type GABA_B1_. Expression of HA-tagged GABA_B_ receptors was determined after 2 days by staining with antibodies directed against the HA-tag. Left: Representative images of total GABA_B_ receptor expression (scale bar: 10 μm). Right: Quantification of GABA_B2_ staining. Expression levels were normalized to the control (wild type HA-GABA_B2_). The data represent the mean ± SD of 87–144 neurons from three independent. Brown–Forsythe and Welch ANOVA with Games Howell’s multiple comparison test (****p < 0.0001). **(B–E)** Overexpression of the catalytic subunit of PP2A (PP2A-C) downregulates total and cell surface expression of GABA_B_ receptors in neurons. Neurons were transfected with EGFP and either PP2A-C or an inactive mutant of PP2A-C (L199P). Cell surface and total endogenous GABA_B1_ **(B,C)** and endogenous GABA_B2_ **(D,E**) was determined after 2 days via staining with GABA_B1_ and GABA_B2_ antibodies. Left: Representative images (scale bar: 5 μm). Right: Quantification of GABA_B_ receptor staining. Signals were normalized to the control (only transfected with EGFP). The data represent the mean ± SD of 87–144 neurons from three independent. Brown–Forsythe and Welch ANOVA with Games Howell’s multiple comparison test (ns, *p* > 0.05, *p < 0.05, ****p < 0.0001).

It was previously shown that dephosphorylation of GABA_*B*2_ at S783 by PP2A is involved in glutamate and NMDA receptor-mediated downregulation of GABA_*B*_ receptors (Terunuma et al., 2010). To confirm that the phosphorylation state of S783 in GABA_*B*2_ regulates GABA_*B*_ receptor expression, we transfected primary cortical neurons with either wild type HA-tagged GABA_*B*2_, a HA-tagged GABA_*B*2_ mutant containing a serine-to-alanine substitution at position 783 (S783A) or with serine-to-aspartate substitution (S783D) to mimic the persistent dephosphorylation and phosphorylation status of the receptor, respectively (Figure 1A). As expected, mimicking dephosphorylation of GABA_*B*_(S783) (S783A) significantly reduced GABA_*B*_ receptor expression, while mimicking phosphorylation (S783D) enhanced GABA_*B*_ receptor expression as compared to neurons transfected with wild type HA-tagged GABA_*B*2_ (Figure 1A).

To confirm that PP2A is involved in the regulation of GABA_*B*_ receptor expression, we transfected cortical neurons with the catalytic subunit of PP2A (PP2A-C, constantly active) or with a dominant negative mutant thereof (L199P) and determined endogenous total and cell surface expression of GABA_*B*1_ and GABA_*B*2_ (Figures 1B–E). Overexpressing PP2A-C reduced total and cell surface expression of both GABA_*B*_ receptor subunits. By contrast, overexpressing the inactive mutant of the catalytic subunit of PP2A (L199P) did largely not affect receptor expression.

These results strengthen previous data indicating that dephosphorylation of GABA_*B*2_(S783) by PP2A is crucial for determining the expression level of GABA_*B*_ receptor (Terunuma et al., 2010).

### Blocking the interaction of PP2A with GABA_*B*_ receptors by an interfering peptide enhances GABA_*B*_ receptor surface expression

**FIGURE 2:**
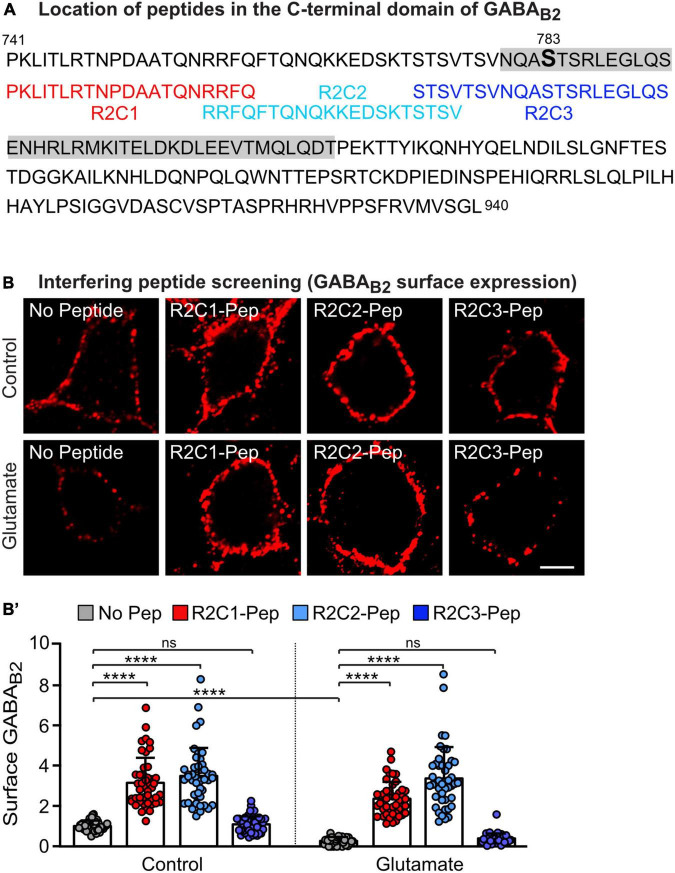
Screening for a peptide sequence present in the C-terminal domain of GABA_B2_ that blocks glutamate-induced downregulation of GABA_B_ receptors. **(A)** Sequence of the C-terminal domain of GABA_B2_ with the coiled-coil domain indicated in gray and the overlapping peptides R2C1, R2C2 and R2C3 used in **(B)** shown below. **(B,B’)** Peptides R2C1 and R2C2 increased expression of GABA_B_ receptors and restored glutamate-induced downregulated receptor cell surface levels. Cultures were stressed for 60 min with glutamate (50 μM) and immediately thereafter treated for 16 h with the peptides indicated in **(A)**. Neurons were then tested for cell surface expression of endogenous GABA_B_ receptors using antibodies directed against the N-terminus of GABA_B2_. **(B)** Representative images of GABA_B2_ cell surface staining (scale bar: 5 μm). **(B’)** Quantification of the fluorescence intensities. The data were normalized to cultures not treated with glutamate and peptides and represent the mean ± SD of 45 neurons derived from three independent experiments. Two-way ANOVA followed by Tukey’s multiple comparison test (ns, *p* > 0.05, ****p < 0.0001.

A prerequisite for the ability of PP2A to dephosphorylate GABA_*B*2_(S783) is its interaction with GABA_*B*_ receptors (Terunuma et al., 2010; Li et al., 2020). To interfere with the PP2A-mediated dephosphorylation of GABA_*B*_ receptors, we aimed to develop a targeted approach to specifically inhibit the PP2A/GABA_*B*_ receptor interaction. Most likely the C-terminal domain of GABA_*B*2_ is involved in constituting the interaction site with PP2A. We therefore screened several small peptide sequences of the C-terminal domain of GABA_*B*2_ for their ability to increase cell surface expression of GABA_*B*_ receptors. The effect of three candidate peptides (R2C1, R2C2, and R2C3) located upstream of the coiled-coil domain of GABA_*B*2_, adjacent or partially overlapping with the S783 phosphorylation site targeted by PP2A (Figure 2A), is shown in Figure 2B. Among these peptides, R2C1 and R2C2 mediated enhancement cell surface GABA_*B*_ receptor expression in cultured neurons under control conditions and after glutamate stress, which downregulated receptor expression (Figures 2B,B’). R2C3 did not affect GABA_*B*_ receptor expression and therefore the sequence of R2C3 appears not to contribute to the GABA_*B*_ receptor/PP2A interaction site. We selected R2C2 for all further experiments because it most reliably increased receptor expression. This peptide (designated PP2A-Pep) was tagged at the N-terminus with a peptide sequence derived from the Rabies virus glycoprotein to render it cell-permeable (Kumar et al., 2007). A control peptide (Ctrl-Pep), consisting of the same amino acids but in a random sequence, was used to verify the specificity of PP2A-Pep in main experiments.

**FIGURE 3:**
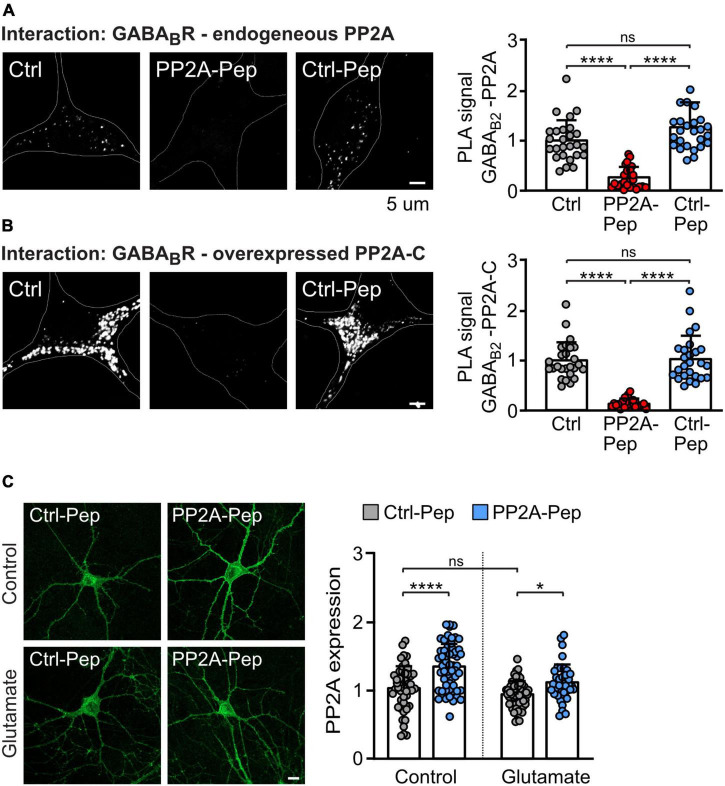
PP2A-Pep inhibits the interaction of GABA_B_ receptors with PP2A and does not downregulate PP2A expression. **(A,B)** PP2A-Pep prevents the PP2A/GABA_B_ receptor interaction. **(A)** The interaction of endogenous PP2A with endogenous total (cell surface and intracellular) GABA_B_ receptors in the presence or absence of PP2A-Pep (10 μM) was analyzed by *in situ* PLA using antibodies directed against PP2A and GABA_B2_. **(B)** The interaction of GABA_B_ receptors and the overexpressed catalytic subunit of PP2A (PP2A-C) in the presence or absence of PP2A or Ctrl-Pep was analyzed by *in situ* PLA. Neurons were co-transfected with EGFP for identification of PP2A-C overexpressing neurons. Neurons transfected only with EGFP and not treated with peptides served as controls. Left: representative images (white dots represent interactions, scale bar: 5 μm). Right: quantification of the *in situ* PLA signals. The data were normalized to control cultures not treated with peptides and represent the mean ± SD of 26 neurons derived from three independent experiments. Brown–Forsythe and Welch ANOVA with Games Howell’s multiple comparison test (ns, *p* > 0.05, *p < 0.05, ****p > 0.0001). **(C)** PP2A-Pep does not downregulate PP2A expression. Glutamate-stressed and unstressed neurons were treated with PP2A-Pep (10 μM) and analyzed for PP2A expression. Left: representative images (scale bar: 10 μm). Right: quantification of the immunofluorescence. The data were normalized to control cultures not treated with glutamate and peptide and represent the mean ± SD of 38–60 neurons derived from three independent experiments. Two-way ANOVA followed by Tukey’s multiple comparison test (ns, *p* > 0.05; *p < 0.05, ****p < 0.0001).

Next, we tested whether PP2A-Pep inhibits the interaction of PP2A with GABA_*B*_ receptors in primary cortical cultures using the *in situ* PLA. We detected PLA signals in neurons, suggesting that PP2A is involved in regulating GABA_*B*_ receptor expression under basal conditions (Figure 3A). The PLA signals were largely inhibited by the treatment with PP2A-Pep, indicating that PP2A-Pep largely prevented the interaction of PP2A with GABA_*B*_ receptors. As expected, the control peptide (Ctrl-Pep) had no effect (Figure 3A).

Furthermore, overexpression of the catalytic subunit of PP2A (PP2A-C) in neurons strongly enhanced PLA signals, indicating an increased interaction of PP2A with GABA_*B*_ receptors (Figure 3B). In addition, this result suggests that the catalytic subunit of PP2A is involved in the interaction with GABA_*B*_ receptors. In contrast to the inactive Ctrl-Pep, PP2A-Pep largely abolished the interaction (Figure 3B). These results indicate that PP2A-Pep prevents the interaction of GABA_*B*_ receptor with PP2A.

Next, we tested whether PP2A-Pep alters the expression of PP2A as it most likely binds to PP2A to prevent its binding to GABA_*B*_ receptors which might induce the degradation of this complex. However, treatment of neurons with PP2A-Pep resulted in a slight (but statistically significant) enhancement of PP2A expression in both control and glutamate stressed neurons, a condition which aberrantly downregulates GABA_*B*_ receptors (Guetg et al., 2010; Maier et al., 2010; Terunuma et al., 2010; Figure 3C).

### PP2A-Pep reduces internalization of GABA_*B*_ receptors under excitotoxic conditions

**FIGURE 4:**
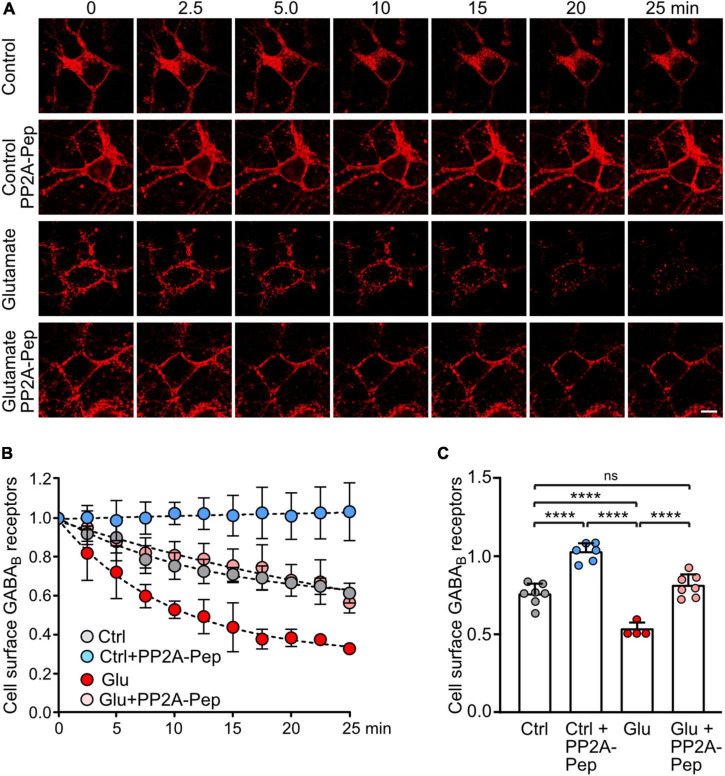
PP2A-Pep prevents constitutive internalization of GABA_B_ receptors and normalizes glutamate-induced loss of cell surface receptors. Neurons were transfected with GABA_B2_ tagged with the binding site for α-bungarotoxin [GABA_B2_(BBS)] along with EGFP for quick identification of transfected neurons and with wild type GABA_B1_. After labeling cell surface receptors containing GABA_B2_(BBS) with AlexaFluor 555 conjugated α-bungarotoxin at 4°C, neurons were analyzed at room temperature by life cell imaging for internalization of labeled receptors at the indicated time points and treatments. **(A)** Representative images (scale bar: 10 μm). **(B)** Quantification of the immunofluorescence signal. **(C)** Statistical evaluation of data 10 min after initiation of internalization. The data represent the mean ± SD of 4–7 neurons derived from three independent experiments. Two-way ANOVA followed by Tukey’s multiple comparison test (ns, *p* > 0.05, ****p < 0.0001).

The dephosphorylation of GABA_*B*2_(S783) by PP2A downregulates receptor expression most likely via aberrant postendocytic sorting after sustained NMDA receptor activation (Terunuma et al., 2010). However, the underlying mechanism is largely unclear. Here, we hypothesized that blocking PP2A-mediated dephosphorylation of GABA_*B*_ receptors using PP2A-Pep enhances cell surface expression by reducing receptor internalization. To test this, we tagged GABA_*B*2_ at the extracellularly located N-terminus with the binding site for α-bungarotoxin [GABA_*B*2_(BBS)], transfected it into cultured neurons and labeled cell surface receptors containing GABA_*B*2_(BBS) with AF555-conjugated α-bungarotoxin followed by live cell imaging with or without glutamate (Figure 4).

Cell surface GABA_*B*_ receptors were reduced to 75 ± 7% of initial receptors 10 min after initiating internalization, indicating rapid internalization of receptors (Figures 4B,C). Treatment of neurons with PP2A-Pep completely prevented loss of cell surface receptors (102 ± 6% of initial receptors), suggesting that PP2A is involved in regulating constitutive endocytosis of GABA_*B*_ receptors. Application of glutamate induced a pronounced reduction in cell surface receptors 10 min after initiating endocytosis (58 ± 4% of initial receptors). Treatment of neurons with PP2A completely prevented aberrant glutamate-induced downregulation of GABA_*B*_ receptors back to normal control conditions (81 ± 7%, Figures 4B,C).

**FIGURE 5:**
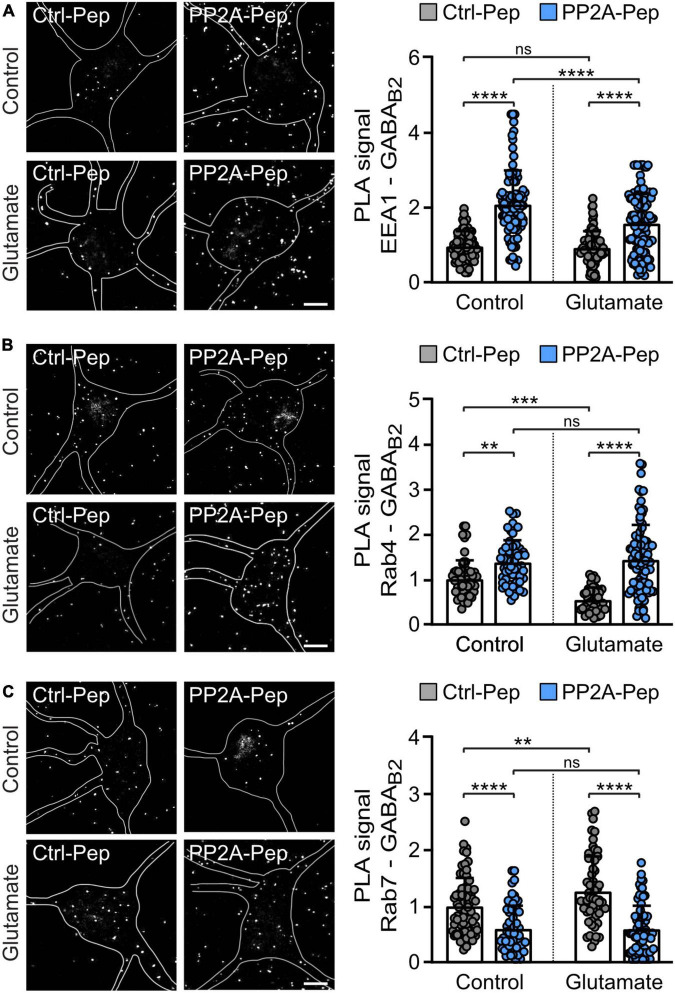
PP2A-Pep increased fast recycling of GABA_B_ receptors and reduced their sorting to lysosomal degradation. Cultured neurons were stressed or not (control) with glutamate (50 μM for 1 h) followed by incubation with PP2A-Pep. Neurons were then analyzed for the colocalization of GABA_B_ receptors with endosomal markers by *in situ* PLA using antibodies directed against GABA_B2_ and EEA1 **(A)**, Rab4 **(B)**, and Rab7 **(C)**. The location and shape of neurons analyzed in the representative images are outlined. Please note that PLA signals outside of the outlined neurons are associated with dendrites of adjacent neurons, which were not marked. **(A)** PP2A-Pep treatment increased the colocalization of GABA_B_ receptors with the early endosome marker EEA1. Left: representative images (scale bar, 10 μm). Right: quantification of PLA signals (white dots). The data represent the mean ± SD of 94–114 neurons per condition from five independent experiments. Two-way ANOVA followed by Tukey’s multiple comparison test (ns, *p* > 0.05; ****p < 0.0001). **(B)** PP2A-Pep treatment increased the colocalization of GABA_B_ receptors with the fast-recycling endosome marker Rab4. Left: representative images (scale bar, 10 μm). Right: quantification of PLA signals. The data represent the mean ± SD of 42–93 neurons per condition from three independent experiments. Two-way ANOVA followed by Tukey’s multiple comparison test (ns, *p* > 0.05, **p < 0.005, ***p < 0.0005, ****p < 0.0001). **(C)** PP2A-Pep treatment reduced colocalization of GABA_B_ receptors with the late endosome marker Rab7. Left: representative images (scale bar, 10 μm). Right: quantification of PLA signals. The data represent as mean ± SD of 58–74 neurons per condition from three independent experiments. Two-way ANOVA followed by Tukey’s multiple comparison test (ns, *p* < 0.05, **p < 0.01, ****p < 0.0001).

To further analyze the pathway underlying PP2A-induced downregulation of GABA_*B*_ receptors, we quantitatively determined the co-localization of the receptors with marker proteins for early endosomes (EEA1) (Mu et al., 1995; Wilson et al., 2000), fast recycling endosomes (Rab4) (van der Sluijs et al., 1992; Daro et al., 1996) and late endosomes (Rab7) (Feng et al., 1995) using *in situ* PLA. Treatment of neurons with PP2A-Pep enhanced colocalization of the receptors with early endosomes and fast recycling endosomes but reduced colocalization with late endosomes (Figure 5). These results suggest that PP2A regulates internalization and fast recycling of the receptors and thereby sorting of the receptors to lysosomal degradation.

Under excitotoxic conditions (treatment of neurons with glutamate), GABA_*B*_ receptors are rapidly downregulated by inhibiting recycling of the receptors and sorting them to lysosomes for degradation (Guetg et al., 2010; Maier et al., 2010; Terunuma et al., 2010). This is reflected by reduced colocalization of the receptors with fast recycling endosomes (Rab4 positive) and enhanced colocalization with late endosomes (Rab7 positive) after treatment of neurons for one hour with 50 μM glutamate (Figure 5). Treatment of glutamate-stressed neurons with PP2A-Pep normalized the aberrant sorting of the receptors back to control levels by restoring fast recycling (colocalization with Rab4-positive endosomes and EEA1-positive endosomes), which reduced lysosomal targeting of the receptors (colocalization with Rab7 positive endosomes) (Figure 5).

### PP2A-Pep-mediated restoration of GABA_*B*_ receptors inhibits excitotoxic neuronal death

**FIGURE 6:**
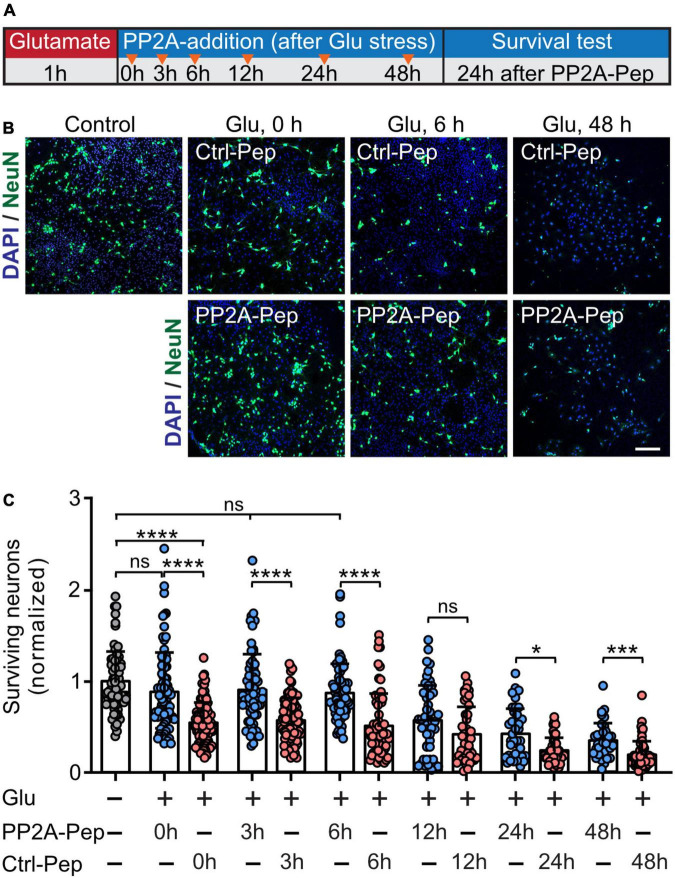
PP2A-Pep inhibits progressing excitotoxic neuronal death. Neurons were stressed for 60 min with 50 μM glutamate and treated with no peptide (control), PP2A-Pep (10 μg/ml) or Ctrl-Pep (10 μg/ml) 0, 3, 6, 12, 24, and 48 h after the glutamate stress. After additional 24 h of incubation, the cultures were stained with DAPI for total cells (glia plus neurons) and with an antibody directed against NeuN for neurons. **(A)** Schematic representation of the experimental design. **(B)** Representative images (scale bar: 100 μm) are shown for the untreated control and for the condition where the peptides were immediately administered after the glutamate stress (0 h), or after 6 and 48 h. **(C)** Quantification of neuronal loss. Number of neurons were normalized to the untreated control cultures. The data represent the mean ± SD of 35–112 frames (fields of view) per experimental condition from three independent experiments. Brown–Forsythe and Welch ANOVA with Games Howell’s multiple comparison test (ns, *p* > 0.05, *p < 0.05, ***p < 0.0005, ****p < 0.0001).

Our previous work showed that stabilization of cell surface GABA_*B*_ receptors by its persistent activation with the agonist baclofen or restoring GABA_*B*_ receptor expression using interfering peptides targeting the interaction of GABA_*B*_ receptors with CaMKII or CHOP, respectively, mediates neuroprotection after ischemic stress *in vitro* and *ex vivo* (Hleihil et al., 2021; Balakrishnan et al., 2022; Bhat et al., 2022). We therefore tested if treatment with PP2A-Pep limits glutamate induced neuronal death. Cortical neurons were stressed for 60 min with glutamate and treated with PP2A-Pep at different time intervals thereafter (0, 3, 6, 12, 24, and 48 h). The number of surviving neurons was determined 24 h after treatment with the peptide at each timepoint. As expected, treatment with PP2A-Pep following glutamate stress inhibited progressing neuronal death at virtually all time points tested (Figure 6). Notably, treatment of neurons with PP2A-Pep 48 h after the excitotoxic stress still limited progressing neuronal death, indicating a very wide time window for the neuroprotective activity of PP2A-Pep. This finding further supports our hypothesis that restoring GABA_*B*_ receptor expression by blocking the PP2A/GABA_*B*_ receptor interaction limits neuronal death under excitotoxic conditions.

### PP2A-Pep provides neuroprotection in a mouse model of ischemic stroke

Our *in vitro* data indicate a potent neuroprotective activity of PP2A-Pep caused by inhibiting the interaction of GABA_*B*_ receptors with PP2A, which restores GABA_*B*_ receptor expression after excitotoxic stress. We therefore tested the effects of PP2A-Pep in the MCAO mouse model of cerebral ischemia. Mice were subjected to 60 min MCAO and the effect of PP2A-Pep was tested on brain slices containing the somatosensory cortex *ex vivo* 6 h after the insult (this time span ensures healthy *ex vivo* brain slices). The somatosensory cortex was selected because it belongs to the ischemic penumbra that is responsive to neuroprotective treatments.

**FIGURE 7:**
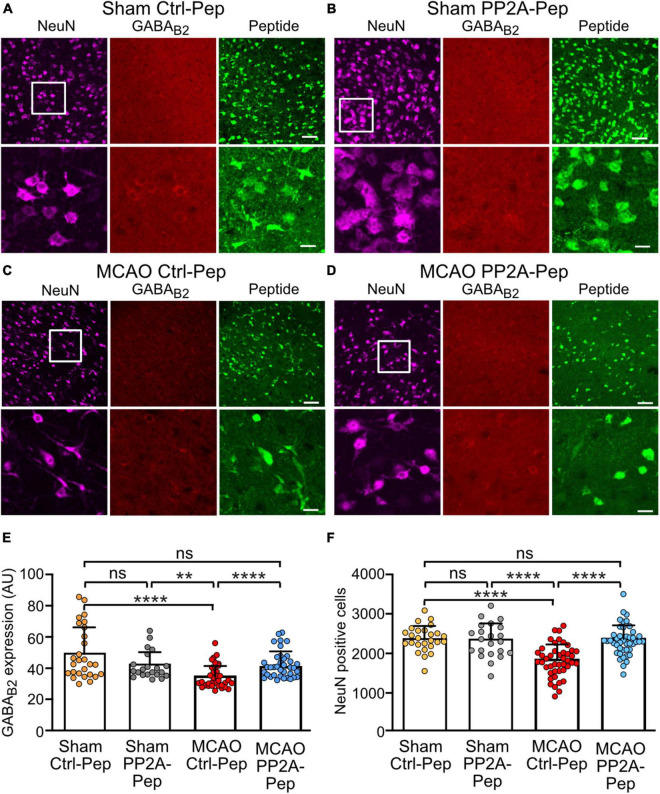
PP2A-Pep normalized receptor expression and inhibited neuronal death after MCAO-induced cerebral ischemia. Brain slices containing the somatosensory cortex were prepared from sham and MCAO operated mice and incubated for 6 h with 10 μg/ml PP2A-Pep followed by staining for GABA_B_ receptors (red), neurons (magenta, NeuN positive cells) or PP2A-Pep (green). Representative images are shown for sections from sham operated mice treated with Ctrl-Pep **(A)** or PP2A-Pep **(B)** and for section from MCAO operated mice treated with Ctrl-Pep **(C)** or PP2A-Pep **(D)**. Images on top of each panel are of low magnification (scale bar: 40 μm) and those on the bottom are of higher magnification (scale bars 10 μm). **(E)** Quantification of GABA_B_ receptor expression. The data represent the mean ± SD of 21–37 frames (fields of view) derived from 4 to 5 mice per condition. Two-way ANOVA with Tukey’s multiple comparison test (ns, *p* > 0.05, **p < 0.005, ****p < 0.0001). **(F)** Quantification of neuronal loss. The data represent the mean ± SD of 22–44 frames (fields of view) derived from 4 to 6 mice per condition. Two-way ANOVA with Tukey’s multiple comparison test (ns, *p* > 0.05, ****p < 0.0001.

Brain slices obtained from MCAO mice treated with Ctrl-Pep exhibited a reduced expression of GABA_*B*_ receptors as compared to sham operated mice treated with Ctrl-Pep or PP2A-Pep as monitored by GABA_*B*2_ antibodies (Figures 7A–C,E). The reduced GABA_*B*_ receptor expression was accompanied by significant neuronal death as monitored by the loss of NeuN positive cells (Figures 7A–C,F). Treatment of slices with PP2A-Pep for 6 h restored GABA_*B*_ receptor expression to normal levels in brain slices of MCAO mice and prevented the loss of neurons (Figures 7D–F). In addition, we observed a robust colocalization of peptide staining with NeuN-positive cells, confirming the predominantly neuron-specific uptake of peptides tagged with Rabis virus glycoprotein sequences (Figure 7). These data illustrate that treatment of *ex vivo* brain slices with PP2A-Pep about 1 h after MCAO restored GABA_*B*_ receptor expression and inhibited progressing neuronal death.

### PP2A-Pep restores GABA_*B*_ receptor function after ischemic stress

**FIGURE 8:**
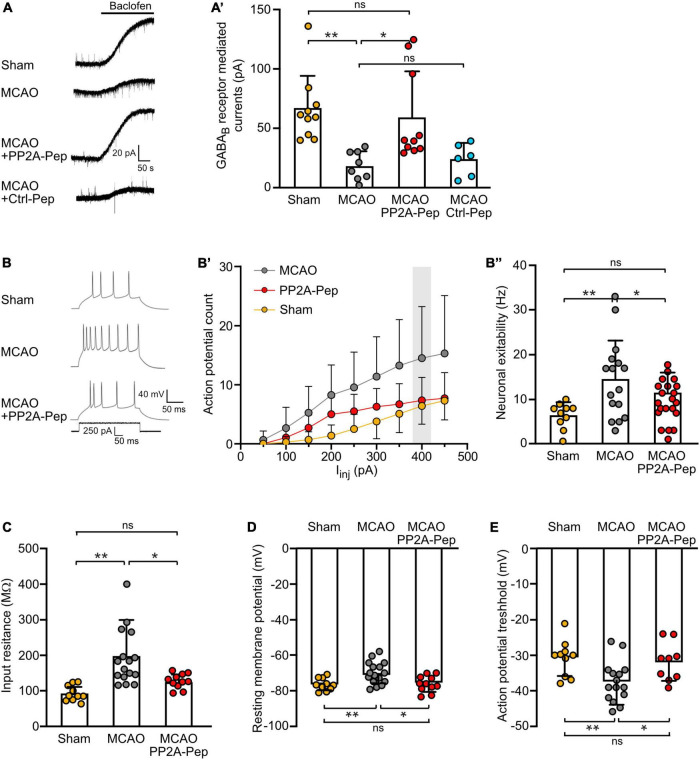
PP2A-Pep restored GABA_B_ receptor-mediated inhibition and reduced neuronal excitability after MCAO-induced cerebral ischemia. Brain slices containing the somatosensory cortex of sham and MCAO operated mice were analyzed using whole-cell patch-clamp recordings 2–6 h after treatment with PP2A-Pep, Ctrl-Pep or saline. **(A)** Representative traces of baclofen-evoked currents. **(A’)** Quantification of baclofen-evoked current traces. The data represent the mean ± SD of 6–10 neurons. One-way ANOVA, Tukey’s multiple comparison test (ns, *p* > 0.05, *p < 0.05, **p < 0.01). **(B–B”)** Neuronal excitability was determined by injecting increasing current steps. **(B)** Representative current traces. **(B’)** Quantification of action potential firing evoked by increasing current injections. **(B”)** Bar graph and statistical evaluation of data depicted in the gray box in **(B’)**. The data represent the mean ± SD of 10–16 neurons. Brown–Forsythe and Welch ANOVA with Games Howell’s multiple comparison test (ns, *p* > 0.05, *p < 0.05, **p < 0.01). **(C)** Determination of input resistance. **(D)** Determination of resting membrane potential. **(E)** Determination of action potential threshold. The data represent the mean ± SD of 10–16 neurons. One-way ANOVA with Tukey’s multiple comparison test (ns, *p* > 0.05, *p < 0.05, **p < 0.01).

Since PP2A-Pep restored GABA_*B*_ receptor expression in brain slices from MCAO-treated mice, we next tested if exposure to PP2A-Pep also restored normal GABA_*B*_ receptor-mediated inhibition using whole-cell patch-clamp recordings. In line with the downregulation of GABA_*B*_ receptors in MCAO brain slices (Figure 7), GABA_*B*_ receptor-mediated currents were strongly reduced (Figures 8A,A’). However, incubation of the slices with PP2A-Pep restored GABA_*B*_ receptor-mediated currents to levels observed in brain slices obtained from sham-operated mice. By contrast, treatment with Ctrl-Pep did not affect GABA_*B*_ receptor-mediated currents in MCAO brain slices, documenting the specificity of the PP2A-Pep effect (Figures 8A,A’).

Since GABA_*B*_ receptor-mediated inhibition is a key player regulating neuronal excitability, we test whether the reduction of GABA_*B*_ receptor-mediated inhibition is accompanied with increased neuronal excitability. As expected, neurons in slices from MCAO mice exhibited enhanced firing frequency as compared to neurons from sham-operated mice (Figure 8B). This was reflected by an enhanced input resistance (Figure 8C), a depolarized resting membrane potential (Figure 8D), and a hyperpolarized threshold for action potential firing (Figure 8E). Incubation of the slices with PP2A-Pep after ischemia normalized the ischemia-induced alterations in passive neuronal properties, resulting in a reduced neuronal firing rate (Figures 8B–E).

These data indicate that blocking the PP2A/GABA_B_ receptor interaction restored normal GABA_B_ receptor inhibition which resulted in reduced neuronal excitability after cerebral ischemia.

## Discussion

In this study, we developed a small interfering peptide that inhibits the interaction of GABA_B_ receptors with PP2A to analyze in more detail the trafficking pathways affected by PP2A and to explore the therapeutic potential of specifically interfering with the GABA_B_ receptor/PP2A interaction using cerebral ischemia as a model for a severely disturbed excitation/inhibition balance.

GABA_B_ receptors are downregulated under pathological conditions by a mechanism involving CaMKII-mediated phosphorylation of GABA_B1_(S867) (Guetg et al., 2010; Zemoura et al., 2019; Balakrishnan et al., 2022) and PP2A-mediated dephosphorylation of GABA_B2_(S783) (Terunuma et al., 2010; Li et al., 2020). This regulation of the GABA_B_ receptor phosphorylation status aberrantly sorts constitutive internalized receptors to lysosomal degradation instead of recycling them back to the plasma membrane. Severely elevated intracellular Ca^2+^ levels by sustained activation of glutamate receptors enhance phosphorylation of GABA_B1_(S867) by CaMKII, which promotes K68-linked ubiquitination of GABA_B_ receptors at multiple sites by the ubiquitin E3 ligase MIB2 and thereby targets the receptors to the ubiquitin-dependent lysosomal degradation pathway (Zemoura et al., 2016). In this pathway, CaMKII acts after the receptors are internalized, most likely at the level of early endosomes where the decision for recycling or degradation of the receptors is made.

Under the same conditions, GABA_B_ receptors are initially phosphorylated by AMPK at GABA_B2_(S783). This appears to be a response to stabilize the receptors at the cell surface (Kuramoto et al., 2007; Terunuma et al., 2010). However, GABA_B2_(S783) then gets dephosphorylated by PP2A, which contributes to the aberrant sorting of the receptors to lysosomal degradation (Terunuma et al., 2010). Both events, phosphorylation of GABA_B1_(S867) and dephosphorylation of GABA_B2_(S783) are key steps in the pathological downregulation of GABA_B_ receptors since blocking CaMKII or PP2A activity with specific inhibitors blocks this detrimental process (Guetg et al., 2010; Terunuma et al., 2010; Zemoura et al., 2019).

To analyze the detailed trafficking pathways affected by PP2A, we identified an interfering peptide (PP2A-Pep) that blocks the GABA_B_ receptor/PP2A interaction by screening a small library of synthetic peptides comprising the intracellularly located C-terminal domain of GABA_B2_. PP2A-Pep is composed of an amino acid sequence located close to S783 of GABA_B2_. PP2A-Pep effectively blocked the interaction of PP2A with GABA_B_ receptors, upregulated cell surface expression of the receptors cultured in neurons under normal physiological conditions and after excitotoxic stress. A PP2A/GABA_B2_ interaction site close to GABA_B2_(S783) is not surprising as this site is the substrate of the dephosphorylating activity of PP2A. Interestingly, Li et al. (2020) identified another interaction site of PP2A in the C-terminal domain of GABA_B1_. As PP2A-Pep, a peptide comprising the amino acid sequence of the GABA_B1_ interaction site inhibited the interaction of PP2A with GABA_B_ receptors (Li et al., 2020). Therefore, it is very likely that PP2A interacts with GABA_B_ receptors at least via two distinct sites for proper dephosphorylation of GABA_B2_(S783).

The number of cell surface receptors is determined by a precise balance of receptor exocytosis, endocytosis, recycling, and degradation. Phosphorylation of GABA_B1_(S867), most likely at the level of early endosomes, serves as a signal for sorting the receptors to late endosomes by promoting K63-linked ubiquitination of the receptors via MIB2 (Zemoura et al., 2019). The data of this study suggests that PP2A-dependent dephosphorylation of GABA_B2_(S783) takes place upstream of CaMKII phosphorylation of GABA_B1_(S867). Inhibition of the GABA_B_ receptor/PP2A interaction in neurons under physiological conditions completely prevented constitutive reduction of cell surface receptors. This suggests that PP2A may regulate endocytosis of the receptors. However, it is very unlikely that GABA_B2_(S783) dephosphorylation directly serves as a signal for receptors endocytosis since in glutamate-stressed neurons PP2A-Pep did not completely block removal of cell surface receptors but restored it to normal levels of constitutive internalization. In addition, our previous findings indicate that glutamate stress, which induces GABA_B2_(S783) dephosphorylation, did not enhance constitutive internalization of GABA_B_ receptor (Maier et al., 2010). Therefore, GABA_B2_(S783) dephosphorylation most likely inhibits recycling of the receptors. This view is in line with our finding that preventing the interaction of GABA_B_ receptor with PP2A resulted in enhanced colocalization of the receptors with EEA1-positive early endosomes and Rab4-positive fast recycling endosomes, which originate from early endosomes (Sheff et al., 1999). The enhanced recycling of the receptors should stabilize the number of receptors at the cell surface. Thus, the most likely scenario for the sequence of events in glutamate-induced downregulation of GABA_B_ receptors is that PP2A-dependent dephosphorylation of GABA_B2_(S783) inhibits recycling of the receptors and subsequently CaMKII-dependent phosphorylation of GABA_B1_(S867) promotes K63-linked ubiquitination of the receptors by MIB2, which then serves as sorting signals for the lysosomal degradation pathway.

A main purpose of this study was to test the therapeutic potential of specifically blocking the interaction of GABA_B_ receptors with PP2A using an interfering peptide. Because PP2A is involved in regulating a multitude of pathways, globally blocking PP2A activity with specific inhibitors is expected to be associated with severe side effects. Therefore, a targeted approach specifically blocking the interaction of PP2A with GABA_B_ receptors might be a valid alternative. Since GABA_B_ receptors are downregulated under excitotoxic/ischemic conditions occurring in cerebral ischemia (Guetg et al., 2010; Maier et al., 2010; Terunuma et al., 2010; Kim et al., 2011; Kantamneni et al., 2014; Zhu et al., 2015; Huang et al., 2017; Hleihil et al., 2021; Balakrishnan et al., 2022; Bhat et al., 2022), we tested the ability of PP2A-Pep to normalize GABA_B_ receptor expression/function and its neuroprotective activity *in vitro* using glutamate-stressed neuronal cultures and in the MCAO mouse model of cerebral ischemia *ex vivo*. In cultured glutamate-stressed neurons, PP2A-Pep normalized GABA_B_ receptor expression and inhibited progressing neuronal death even when applied 48 h after the insult. This is a very promising finding in view of the development of a therapeutic application for inhibiting progressing neuronal death in acute stroke patients. Most importantly, application of PP2A-Pep to brain slices prepared from MCAO-treated mice restored GABA_B_ receptor expression and function to normal levels, reduced ischemia-induced over-excitability of neurons, and prevented neuronal death in the ischemic penumbra, which is the main target for neuroprotection. Unfortunately, we could test PP2A-Pep in MCAO-treated mice only *ex vivo* because of the poor pharmacokinetics of the current peptide. This limited the time for analysis to 6 hours after the insult. Future optimization of PP2A-Pep for systemic application *in vivo* is required to explore its full neuroprotective potential *in vivo*.

These promising results mirror our recent findings targeting the CaMKII/GABA_B_ receptor interaction, which is involved in this pathway apparently downstream of PP2A-dependent dephosphorylation of GABA_B2_(S783) (Balakrishnan et al., 2022). In addition to the glutamate-induced aberrant lysosomal sorting of GABA_B_ receptors involving CaMKII and PP2A, ischemic conditions further cause downregulation of cell surface receptors via interaction with the pro-apoptotic transcription factor CHOP (Maier et al., 2014). The severe endoplasmic stress associated with ischemic conditions upregulates CHOP, which, in addition to inducing apoptosis, interacts with GABA_B_ receptors in the ER and prevents their export to the cell surface. Inhibition of the CHOP/GABA_B_ receptor interaction with an interfering peptide also normalized GABA_B_ receptor expression after ischemic stress, reduced stress-induced neuronal overexcitation and neuronal death in cultured neurons (Bhat et al., 2022). Since interfering with any of these detrimental GABA_B_ receptor interactions restored GABA_B_ receptor-inhibition and eventually inhibited progressing neuronal death, it would be very interesting to test the neuroprotective activity of a combination of all three interfering peptides targeting the distinct trafficking events in this pathway downregulating GABA_B_ receptors. In addition, it would be interesting to analyze whether the GABA_B_ receptor agonist baclofen can potentiate the neuroprotective activity of PP2A-Pep. In a recent study we showed that baclofen stabilizes GABA_B_ receptor expression under ischemic conditions by enhancing fast recycling of the receptors (Hleihil et al., 2021). If GABA_B_ receptors in the ischemic penumbra are not fully activated by enhanced release of GABA, co-treatment of PP2A-Pep with baclofen is expected to exert a higher neuroprotective activity than treatment of PP2A or baclofen alone.

Apart from cerebral ischemia, which is an extreme pathological situation, interfering with the GABA_B_ receptor/PP2A interaction might also be effective for the treatment of less severe neurological diseases associated with downregulation of GABA_B_ receptors and a disturbed excitation/inhibition balance such as addiction, anxiety, and depression (Maeda et al., 2006; Padgett et al., 2012; Hearing et al., 2013; Lecca et al., 2016). Therefore, targeting the GABA_B_ receptor/PP2A interaction is a promising strategy for the development of a novel therapeutic intervention.

## Data availability statement

The raw data supporting the conclusions of this article will be made available by the authors, without undue reservation.

## Ethics statement

The animal study was reviewed and approved by Zurich cantonal veterinary office, Zurich, Switzerland (license: ZH152/16, ZH011/19, and ZH031/16).

## Author contributions

MH and KB performed the experimental work and analyzed the data. MH, KB, and DB designed the study. DB supervised the experimental work and analyzed the data. MH and DB wrote the manuscript. All authors discussed the results and commented on the manuscript.
